# Supplementary material for: Aliens on the Road: Surveying Wildlife Roadkill to Assess the Risk of Biological Invasion
Source: Biology (Basel). 2023 Jun 13;12(6):850. doi: 10.3390/biology12060850 (PMC10294960; doi:10.3390/biology12060850)
Supplement: Supplementary file 1 [file biology-12-00850-s001.zip › SupplementaryMaterialS3.pdf]

Supplementary material S3 of the article:

Aliens on the road: surveying wildlife road-kills for assessing the risk of biological invasion

Andrea Viviano, Marcello D'Amico, and Emiliano Mori

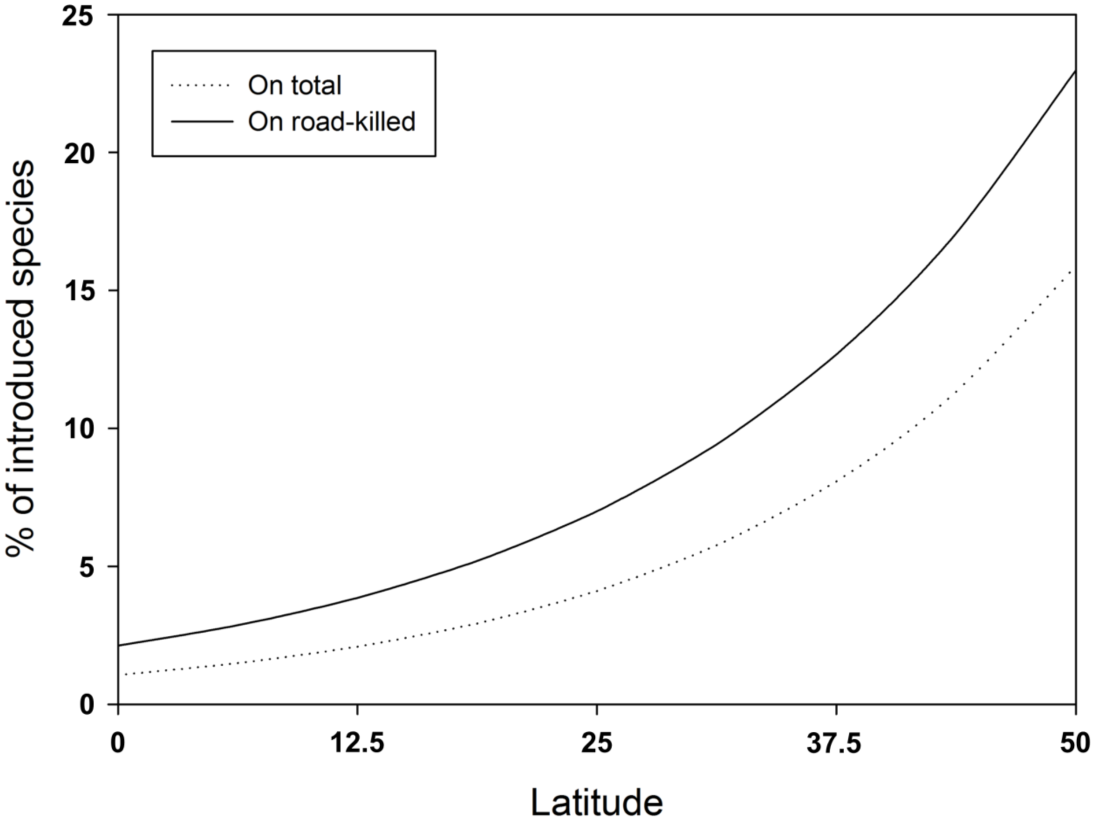

Figure S3. Relation between the *percentage of introduced species* (both *on the total of road-killed species* and *on the total of existing species*) and the latitude of the country hosting the study areas.
