# Supplementary material for: Aliens on the Road: Surveying Wildlife Roadkill to Assess the Risk of Biological Invasion
Source: Biology (Basel). 2023 Jun 13;12(6):850. doi: 10.3390/biology12060850 (PMC10294960; doi:10.3390/biology12060850)
Supplement: Supplementary file 1 [file biology-12-00850-s001.zip › SupplementaryMaterialS4.pdf]

Supplementary material S4 of the article:

Aliens on the road: surveying wildlife road-kills for assessing the risk of biological invasion

Andrea Viviano, Marcello D'Amico, and Emiliano Mori

Table S4. The *percentage of introduced species on the total of road-killed species*, differences among the countries included in the Mediterranean biome, model ranks by AICs. K is the number of model parameters. ΔAICc is the relative difference of a given AIC value compared to the smallest AICc value. AICc weights (wAICc) indicate the relative support for every model (the weights of all the models in the candidate set have the sum of 1). Evidence ratio (ER) is the ratio of wAIC, comparing the best supported model with every competing one. R^2 is a measure of the global fit of each model.

Model	K	AICc	ΔAICc	wAICc	ER	Rank	R^2
Country	5	40.6	0.0	0.79	1.0	1	86.9
Null	2	43.2	2.6	0.21	3.7	2	0.00
